# Local injection of infliximab into calcinosis lesions in patients with juvenile dermatomyositis (JDM): a clinical trial

**DOI:** 10.1186/s12969-023-00941-5

**Published:** 2024-01-02

**Authors:** Reza Shiari, Mitra Khalili, Vahide Zeinali, Niloufar Shashaani, Mohammad Samami, Foroughossadat Hosseini Moghaddamemami

**Affiliations:** 1 Research Institute for Children’s Health, Shahid Beheshti University of Medical Sciences, Tehran, Iran; 2 Dental Sciences Research Center, Department of Oral and Maxillofacial Medicine, School of Dentistry, Guilan University of Medical Sciences, Rasht, Iran; 3 Department of Pediatric Rheumatology, School of Medicine, Guilan University of Medical Sciences, Rasht, Iran

**Keywords:** Juvenile dermatomyositis, Calcinosis, Infliximab, Injections, Intralesional

## Abstract

**Background:**

Juvenile Dermatomyositis (JDM) is a rare autoimmune disorder that primarily affects muscles and skin. One of the severe complications associated with JDM is calcinosis, and treating this condition presents significant challenges. This study aimed to evaluate the efficacy and safety of local injection of infliximab into calcinosis lesions in patients with JDM.

**Methods:**

In this clinical trial, five patients diagnosed with JDM and calcinosis lesions were enrolled. The primary treatment consisted of weekly infliximab injections for 16 weeks, targeting all four sides of each lesion. Lesion dimensions, including length and width, were documented and monitored weekly. Before the intervention, patients underwent radiographic imaging. After the final injection in week 16, a follow-up radiographic assessment was performed. Data were analyzed using the Generalized Estimating Equation (GEE) method.

**Results:**

The lesions’ size significantly decreased in both length and width during each visit. On average, the lesion length reduced by 2.66%, and the width shrank by 3.32% per visit. Based on radiographic findings, the average length and width of lesions at the initial visit were 12.09 ± 5.05 mm (range: 6.00-25.50 mm) and 6.35 ± 3.00 mm (range: 2.00–16.00 mm), respectively. The average length and width at the last visit were 5.59 ± 7.05 mm (range: 0–23.00 mm) and 3.41 ± 4.05 mm (range: 0–13.00 mm), respectively. No specific side effects related to the treatment were reported.

**Conclusions:**

The results suggest that the direct administration of infliximab into the calcinosis lesions of patients with JDM could be a safe and effective treatment approach.

**Trial registration:**

Name of the registry: The effect of infliximab injection into calcinosis lesions on patients with juvenile dermatomyositis (JDM), Trial registration number: IRCT20210808052107N1, Registration date: 2022-07-22, URL of trial registry record: https://en.irct.ir/trial/58329.

**Supplementary Information:**

The online version contains supplementary material available at 10.1186/s12969-023-00941-5.

## Background

Juvenile dermatomyositis (JDM) is a rare autoimmune disease of childhood and the most common type of juvenile idiopathic inflammatory myopathy mainly affecting the skin and muscles [1]. JDM has an annual incidence of 2–4 cases per 1 million children [2, 3]. The precise cause of the disease remains unclear, but it is believed to involve a mix of genetic predisposition and environmental factors. These elements contribute to immune system imbalances and dysfunction, leading to the blood vessel issues seen in JDM. Ultimately, this results in inflammation and tissue damage [4, 5].

Calcinosis is one of the acute and debilitating complications of JDM, which can lead to skin ulcers, joint contractures, muscle atrophy and calcinotic infections [6]. It is defined as the intracellular deposition of insoluble calcium salts in affected tissues, such as the skin, subcutaneous tissue, fascia, tendons and muscles [7]. Approximately 40% of JDM patients experience calcinosis, and unlike adult-onset dermatomyositis, calcinosis in JDM may indicate ongoing disease activity [8]. Managing and treating calcinosis lesions is challenging, with few successful treatments reported [9, 10].

High levels of tumor necrosis factor (TNF) have been reported in patients with JDM who have experienced prolonged disease activity and calcinosis [11–13]. Therefore, anti-TNF drugs, which have proven effective in treating most chronic inflammatory diseases, may be effective on these patients as well [14, 15].

Infliximab is a monoclonal antibody known as a TNF inhibitor in drug classifications. The effect of its systemic injection on patients with JDM has been investigated in limited studies [16–18]. However, in any of the previous studies, infliximab was not injected directly into calcinosis lesions. Encouraged by the positive outcomes of local infliximab injections for the treatment of intestinal strictures in patients with Crohn’s Colitis, as well as the absence of acute complications in a 10-month follow-up period [19–21] we were inspired to utilize this method by injecting infliximab directly into the lesion site. The aim of this study was to evaluate the efficacy and safety of intralesional injection of infliximab in patients with JDM.

## Methods

### Patients

Five patients with JDM and calcinosis lesions participated in a non-randomized, open-label, uncontrolled pilot study. Rigorous inclusion criteria were applied to ensure the safety of injections, restricting participation to patients with calcinosis lesions situated at a considerable distance from vital organs. Patients with lesions larger than 5 cm² were excluded from the study. All patients initially received treatment with IVIG, systemic corticosteroids, hydroxychloroquine, methotrexate, intravenous pamidronate, and systemic biological drugs. None of the patients had a history of receiving topical medication for calcinosis lesions.

### Intervention and monitoring

For each 1.5 cm² calcinosis lesion, 20 mg of infliximab was injected into four points of the lesion. In instances where the lesion size ranged from 1.5 to 5 cm², the infliximab dosage was augmented to 40 mg. The dosage determination was conducted on a per-lesion basis. Local anesthesia was administered to mitigate injection-associated discomfort. Infliximab was diluted for injection and administered into the four sides of the lesion using an insulin syringe. For each lesion, the needle was inserted into the lesion from each of the four sides and infliximab was injected into the lesion. The injection regimen spanned a weekly application over a period of 16 weeks. Radiographic assessment of the lesions occurred before the initiation of the intervention and subsequent to the final injection in the 16th week. Owing to potential hazards linked to radiation exposure, radiography of patients was conscientiously avoided during each visit. Rheumatologists, acting as evaluators, meticulously measured and documented the length and width of the lesions at each visit. To enhance precision and minimize the prospect of errors, two independent rheumatologists conducted measurements, recording the maximum length and width. In cases where disparities in recorded measurements emerged, a re-measurement protocol was enacted, involving the collaboration of a third rheumatologist.

### Statistical analysis

Data were analyzed using SPSS version 23, and a significance level of 5% (P-value < 0.05) was applied. To assess the changes in lesion length and width over a 16-week period, the Generalized Estimating Equation (GEE) was employed.

### Ethical considerations

The study was approved by the Research Ethics Committee of Shahid Beheshti University of Medical Sciences (IR.SBMU.MSP.REC.1401.117). Furthermore, the study protocol was registered and confirmed in the Iranian Registry of Clinical Trails (IRCT20210808052107N1). Written informed consent was acquired from the parents of all participating patients.

## Results

**Fig. 1:**
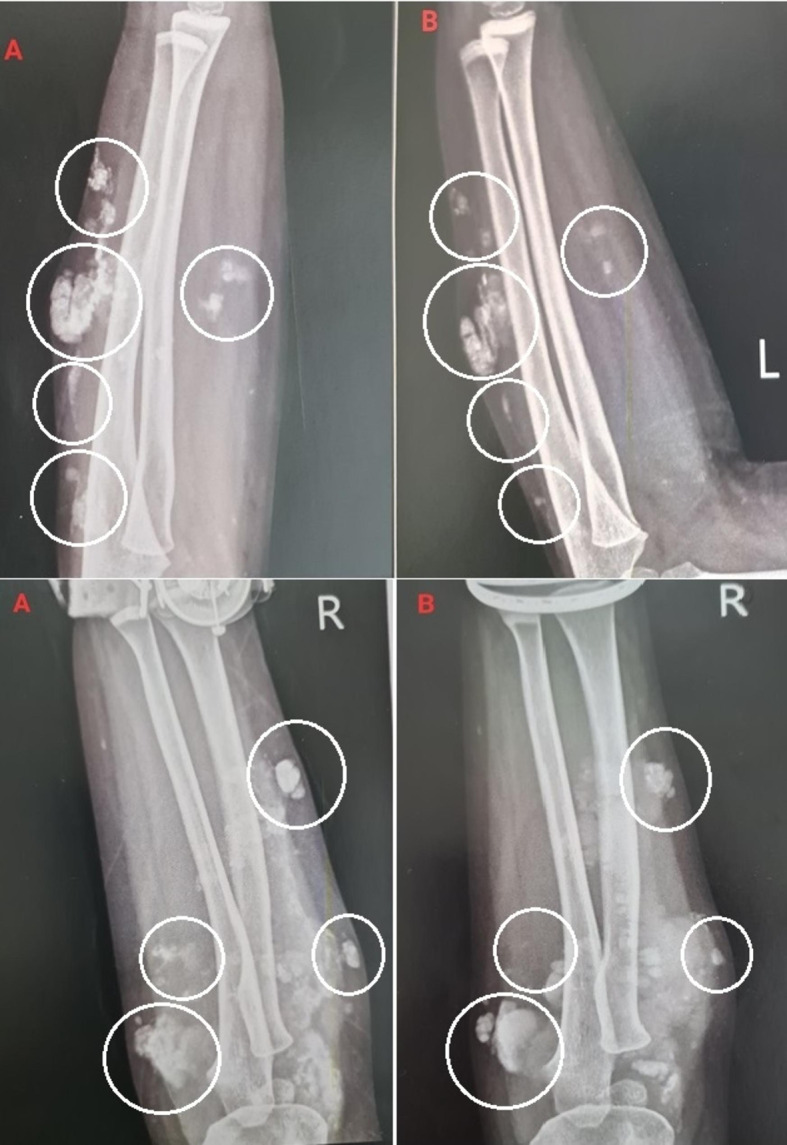
The calcinosis lesions on the left and right forearms of patient 1 before (**A**) and after (**B**) the interventions

Five patients, ranging in age from 5 to 13 years, received local injections of infliximab to treat calcinosis lesions. These lesions commonly developed on the patients’ elbows, hands, and feet. Patient 1, a five-year-old girl, had nine calcinosis lesions on her left and right forearms. Radiographic findings revealed that the injections led to a reduction in lesion size over a period of 16 weeks (Fig. 1).

**Fig. 2:**
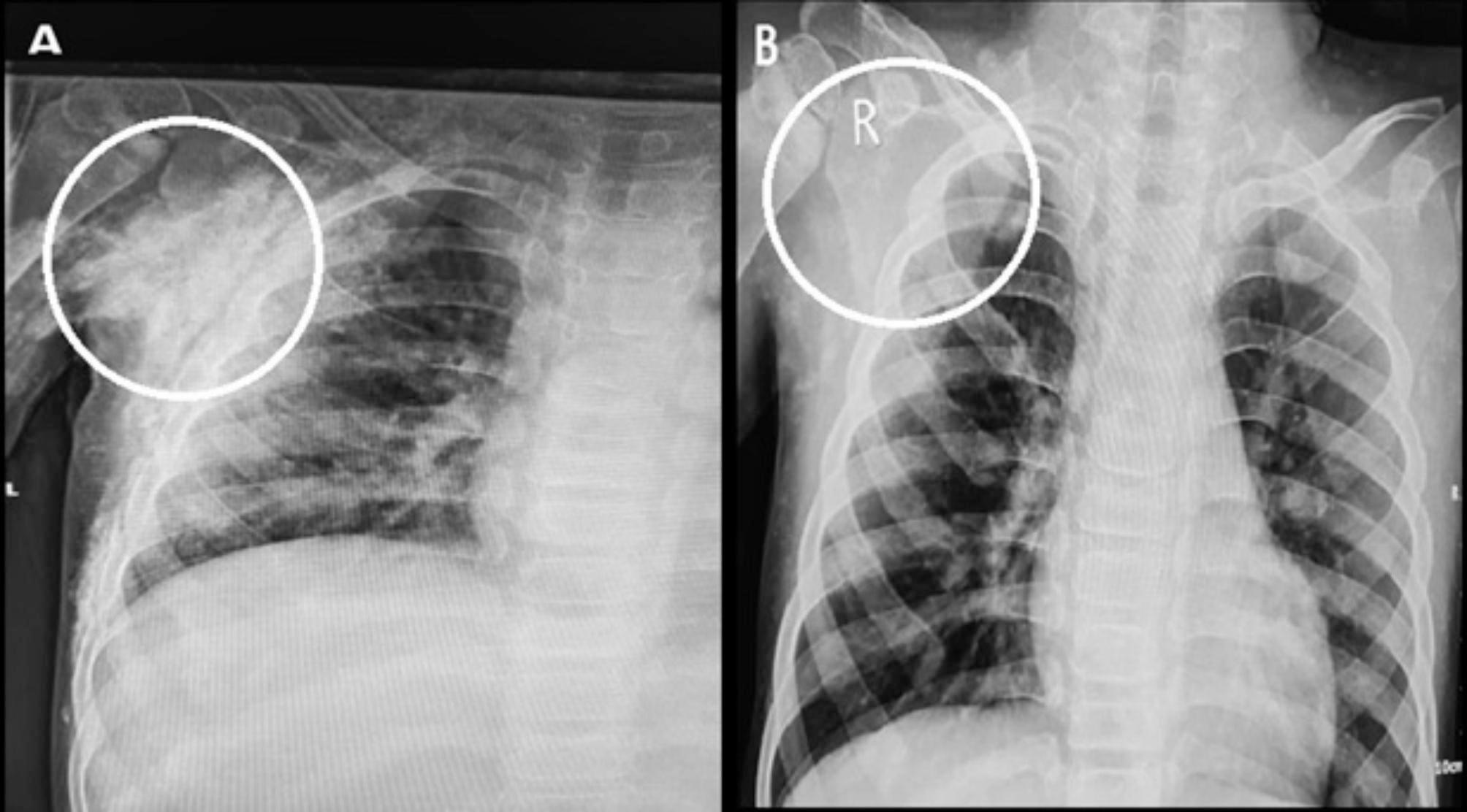
The calcinosis lesion in the axilla of patient 2 before (**A**) and after (**B**) the interventions

Patient 2 was a five-year-old girl who had been suffering from JDM for 17 months. She had a calcinosis lesion (45 × 45 mm) in her axilla, which consisted of several smaller lesions. Due to the limited shoulder abduction, it was challenging to measure the lesion during all visits. As a result, measurements were only taken during the first and last appointments. By the 16th week, the lesion had completely disappeared, and the girl was able to raise her hand above her head (Fig. 2).

**Fig. 3:**
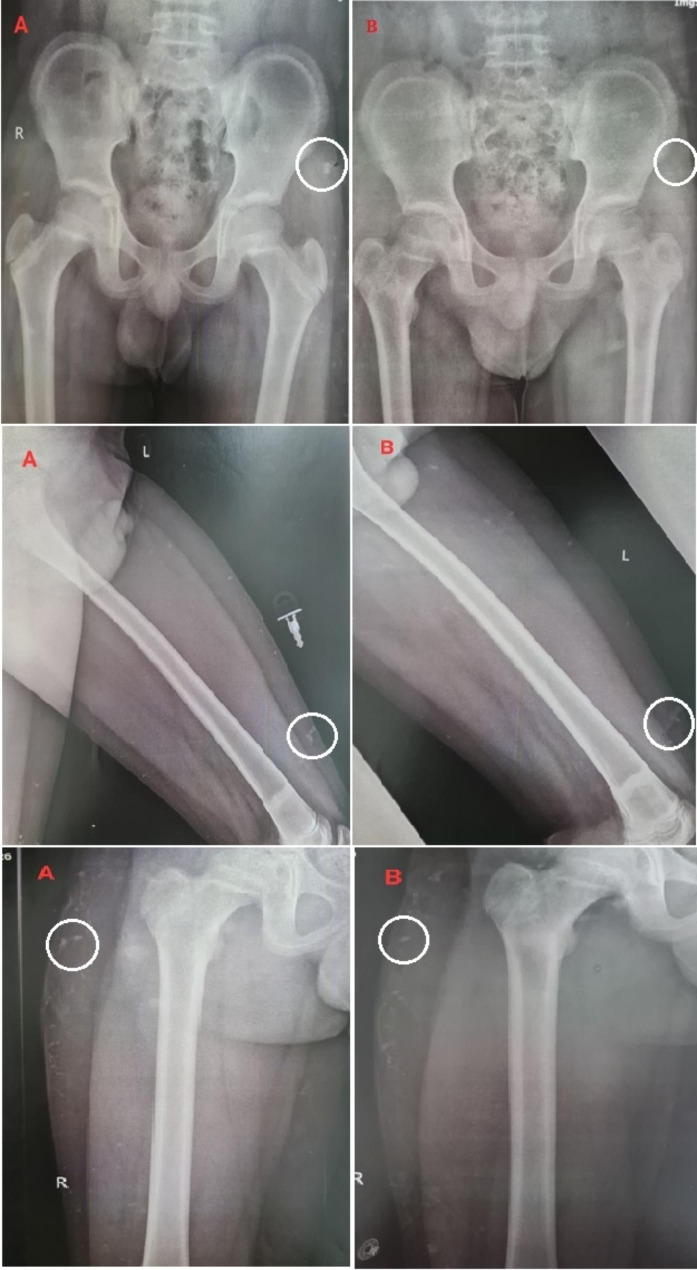
The calcinosis lesions in the lateral pelvic area, left front thigh, and right lateral thigh of patient 3 before (**A**) and after (**B**) the interventions

Patient 3 was a 12-year-old boy who had three calcinosis lesions located in the lateral pelvic area, left front thigh, and right lateral thigh. He had been living with the disease for four years (Fig. 3).

**Fig. 4:**
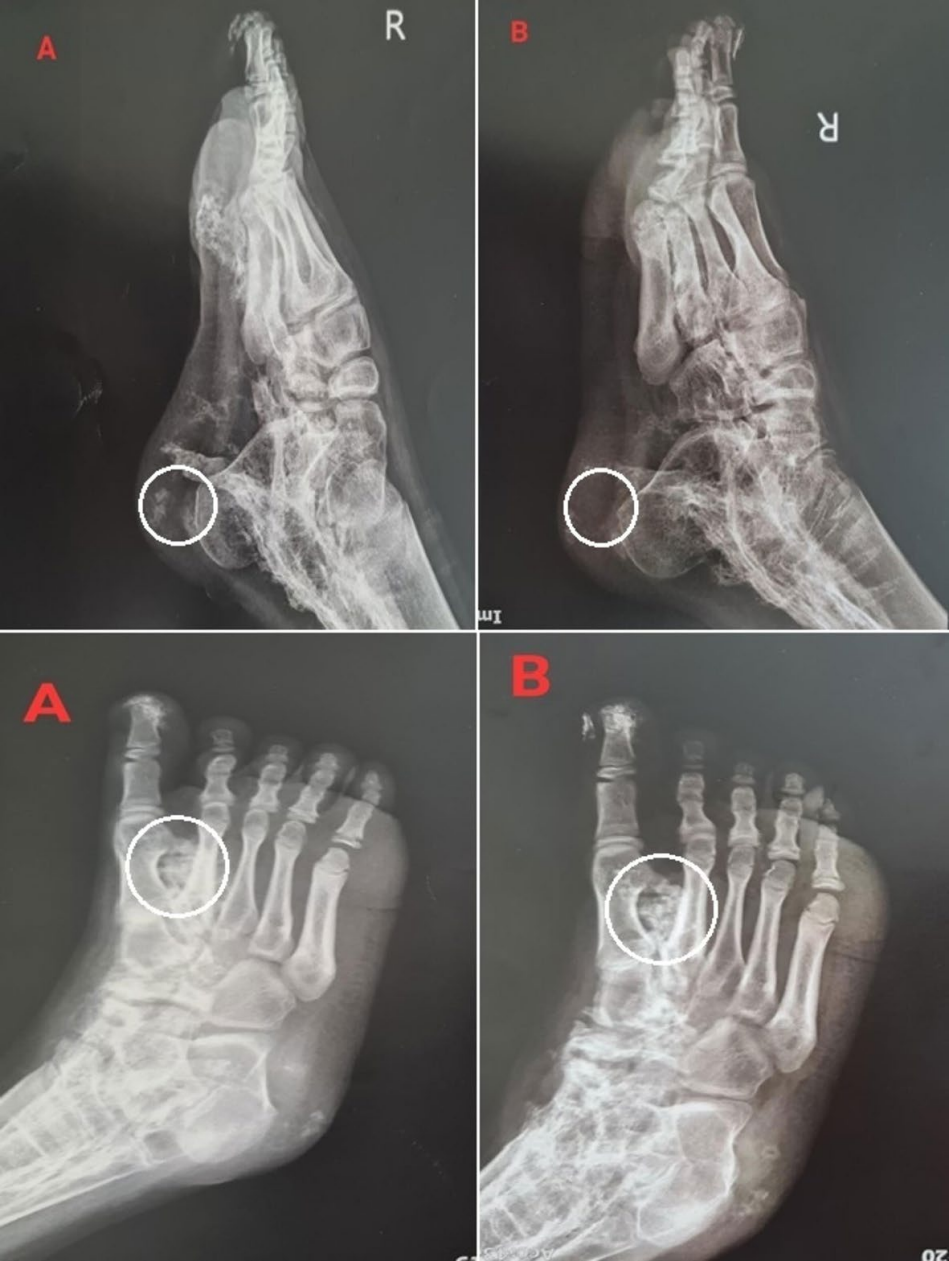
The calcinosis lesions in the heel and sole of the right foot of patient 4 before (**A**) and after (**B**) the interventions

Patient 4, an 11-year-old boy with a 5-year history of JDM, had three calcinosis lesions located on the heel and sole of his right foot (Fig. 4).

**Fig. 5:**
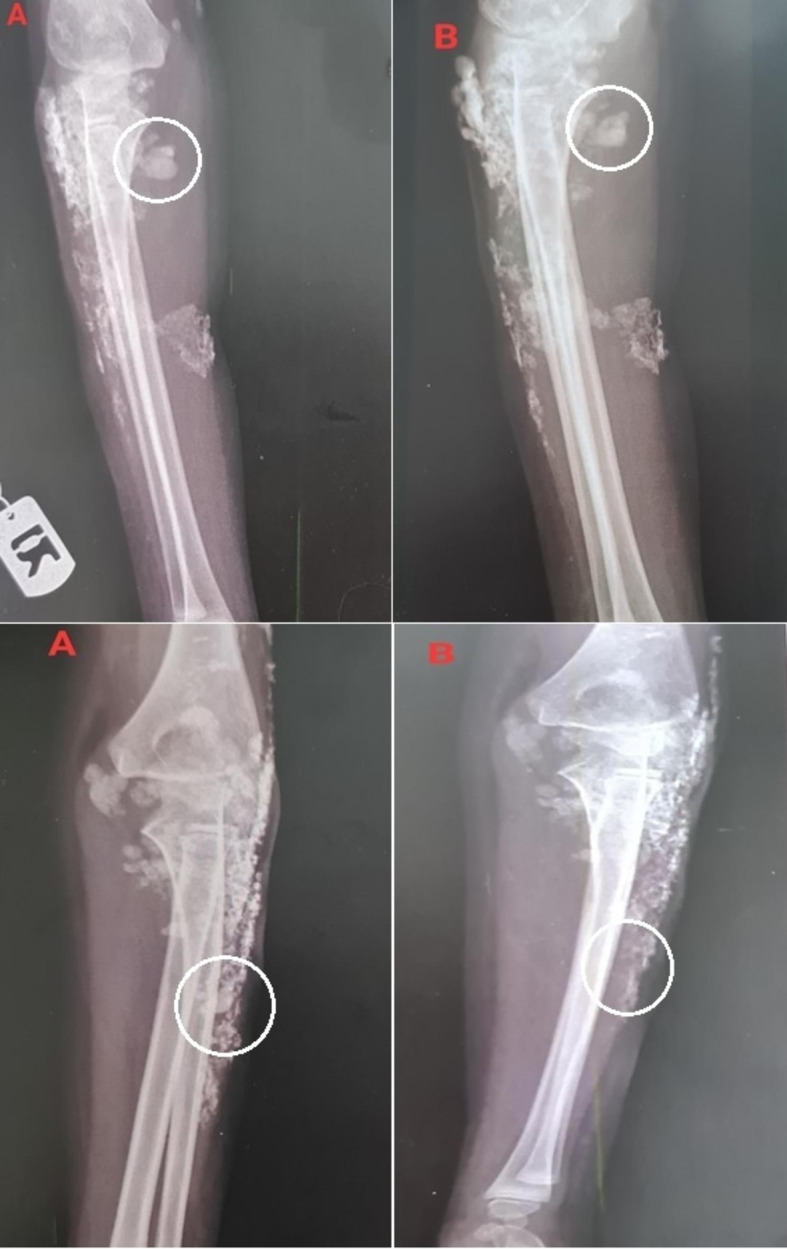
The calcinosis lesions on the left and right forearms of patient 5 before (**A**) and after (**B**) the interventions

Patient 5 was a 13-year-old boy who had two calcinosis lesions on his left and right forearms. He had been living with JDM for nine years (Fig. 5).

The dimensions of calcinosis lesions were documented during each visit (Supplementary Fig. 1). The analysis reveals a significant reduction in the size of the lesions (both length and width) during the treatment process. On average, the length of lesions decreased by 2.66%, while the width of lesions saw a 3.32% reduction per visit (Table 1).

**Table 1:** The percent changes in calcinosis lesions’ dimensions during 16 visits

Lesions’ Length	Coefficient	Std. error.	z	P>|z|	[95% conf. interval]	
Visit time	-2.65769	0.80783	-3.29	0.001	-4.24101	-1.07438
Lesions’ Width						
Visit time	-3.32236	0.949099	-3.5	0.000	-5.18256	-1.46216

Based on radiographic findings, the average length and width of lesions at the initial visit were 12.09 ± 5.05 (6.00-25.50) mm and 6.35 ± 3.00 (2.00–16.00) mm, respectively. By the 16th week, the dimensions of calcinosis lesions had decreased, with the average length and width measuring 5.59 ± 7.05 (0–23.00) mm and 3.41 ± 4.05 (0–13.00) mm, respectively (Supplementary Fig. 2). Throughout the course of the study, there were no reported side effects attributed to the treatment in any of the participating patients.

## Discussion

Calcinosis lesions typically manifest in areas of the body that experience high pressure, including the elbows, knees, forearms, metacarpophalangeal and interphalangeal joints. However, these lesions can appear anywhere throughout the body [22]. In our study, calcinosis lesions frequently presented in the patients’ elbows, forearms, and feet. Notably, only one patient exhibited a lesion in the armpit and axillary region. The duration of JDM experience in our patients was varied: Two patients had JDM for less than two years, while the others had it for four, five, and nine years, respectively. The lesions usually begin one to three years after the onset of dermatomyositis, although some studies have reported cases occurring as late as 20 years after the disease’s onset [23, 24]. In adult dermatomyositis, calcinosis occurs later and is less prevalent than in JDM, with an average onset of 7.8 years compared to 2.9 years and a prevalence of approximately 20% [22].

Various therapeutic approaches have been employed to address calcinosis lesions in JDM patients, such as anti-inflammatory medications and drugs that inhibit calcium metabolism [25]. Nonetheless, there is no established treatment method derived from clinical trials with consistent results. Research on calcinosis lesions reveals the presence of TNF, interleukin-1 beta, and anti-inflammatory cytokines in the calcium milk extracted from these lesions [26]. Given the association between calcinosis development and the TNF-α-308 A polymorphism, it appears that TNF-blocking drugs may be effective in treating calcinosis.

Infliximab is a TNF-blocking drug that has been researched and proven effective for patients with JDM and DM [16, 17, 27]. In a study by Campanilho et al., using two TNF-blocking drugs (infliximab and adalimumab) reduced the number or size of calcinosis lesions in 54% of patients [16]. Additionally, a case series study on five patients with JDM who had calcinosis lesions revealed that administering infliximab at a dosage of 3 mg/kg during the first, second, and sixth weeks, followed by every eight weeks, led to symptom improvement. All patients in their study experienced improvements in joint contractures, calcinosis, and muscle weakness between 8 and 30 months after initiating infliximab treatment. However, calcinosis lesions persisted in four patients, although the lesions became softer and less painful [18].

In light of the favorable outcomes from systemic infliximab injections, as well as the positive impact of localized administration of this drug for treating intestinal strictures in Crohn’s disease patients, this clinical trial involved direct injection of infliximab into calcinosis lesions in JDM patients. Injections were carried out weekly for up to 16 weeks, targeting four areas surrounding each lesion. Upon evaluating the overall changes in the size of calcinosis lesions in the five patients studied, it was observed that the direct injection of infliximab into the lesions led to a significant reduction in both the length and width of these lesions. More specifically, the length of lesions decreased by 2.66% and their width by 3.32% with each injection.

Upon examining the changes in lesion size among the patients, an intriguing observation emerged, although not statistically verifiable in this study. Patients 1 and 2, who had been diagnosed with JDM for less than 2 years, responded more effectively and rapidly to our intervention compared to the other three patients. Patient 2, with a 17-month JDM duration, had a 45 × 45 mm lesion that completely vanished after completing treatment. In Patient 1, with an 18-month JDM duration, the consistent reduction in lesion size began around the fourth injection session and continued until the sixteenth session. Other patients, who had a longer JDM duration, experienced less effective and slower treatment results. Patient 3, with a 4-year JDM duration, exhibited a slow reduction in lesion size with a minimal slope of change. Patient 4, with a 5-year JDM duration, had an even slower reduction process; during several consecutive visits, no change in lesion size was observed, resulting in a flat slope of change on the graph. A similar pattern was seen in patient 5, who had been diagnosed with JDM for 9 years.

Given the association between TNF-α-308 A levels and the disease’s long duration (≥ 36 months) in patients [26], it is reasonable to observe that in two patients with a duration of less than two years, TNF levels are lower than in other patients. Consequently, administering a TNF blocker (infliximab) to the lesion has led to a better and faster response. It appears that the treatment method used in the current study is more effective in patients who have been dealing with JDM for a shorter period. However, to draw a definitive conclusion with strong statistical support, further studies with larger sample sizes are needed. The satisfaction of all five patients with the treatment method and their preference to continue with this approach instead of the conventional drug injection is another noteworthy observation, as reported by the patients and their parents during 16 visit sessions.

Our study had significant limitations, as it was conducted with a limited number of participants, employed an open-label approach, and lacked the inclusion of a control group. The rarity of patients with JDM and calcinosis prevented us from studying an optimal number of patients alongside a control group.

## Conclusions

The findings of the current investigation contribute to reinforcing the proposition that the administration of infliximab through local injection into calcinosis lesions may be efficacious in diminishing the size of such lesions. Drawing definitive conclusions in this domain necessitates further comprehensive research involving a larger sample size. Additionally, by evaluating additional variables such as TNF serum levels and the amount of TNF in the calcium deposits found in the lesions, researchers can identify the factors influencing and predicting the outcomes of this treatment method more precisely.

### Electronic supplementary material

Below is the link to the electronic supplementary material.

Supplementary Material 1

## Data Availability

The data that support the findings of this study are available on request from the corresponding author.

## List of abbreviations

JDM: Juvenile Dermatomyositis
GEE: Generalized Estimating Equation
TNF: Tumor Necrosis Factor
